# Retinal neuronal loss and progression independent of relapse activity in multiple sclerosis

**DOI:** 10.1007/s00415-025-13185-y

**Published:** 2025-06-10

**Authors:** Federico Burguet Villena, Nuria Cerdá-Fuertes, Lisa Hofer, Sabine Schädelin, Shaumiya Sellathurai, Kean Schoenholzer, Marcus D’Souza, Johanna Oechtering, Henner Hanssen, Konstantin Gugleta, Alessandro Cagol, Cristina Granziera, Ludwig Kappos, Axel Petzold, Paskal Benkert, Jens Kuhle, Athina Papadopoulou

**Affiliations:** 1 Department of Neurology, University Hospital Basel, Basel, Switzerland; 2 Department of Clinical Research, University Hospital and University of Basel, Basel, Switzerland; 3 Research Center for Clinical Neuroimmunology and Neuroscience Basel (RC2 NB), University Hospital and University of Basel, Basel, Switzerland; 4 Translational Imaging in Neurology (ThINK) Basel, Department of Biomedical Engineering, Faculty of Medicine, University of Basel, Basel, Switzerland; 5 Neuroimmunology Unit and Multiple Sclerosis Centre, University Hospital of Basel, Basel, Switzerland; 6 Neurostatus AG, University Hospital of Basel, Basel, Switzerland; 7 Department of Sport, Exercise and Health, Medical Faculty, University of Basel, Basel, Switzerland; 8 Department of Ophthalmology, University Hospital of Basel, Basel, Switzerland; 9 Queen Square Institute of Neurology, University College London, London, WC1 N 3BG UK; 10 Dipartimento Di Scienze Della Salute, Università Degli Studi Di Genova, Genova, Italy

**Keywords:** PIRA, PIRMA, OCT, RNFL, GCIPL, Ganglion cells, Neurodegeneration, Multiple sclerosis

## Abstract

**Background:**

In multiple sclerosis (MS), inner retinal thinning measured by optical coherence tomography (OCT) is related to lesional and gray matter changes of the brain.

**Objective:**

To evaluate the association between OCT markers and progression independent of relapse activity (PIRA).

**Methods:**

Analysis within the Swiss MS Cohort Study, in patients with ≥ 1 OCT. Mean thicknesses of: peripapillary retinal nerve fiber— (pRNFL), macular ganglion cell-inner plexiform— (mGCIPL), and inner nuclear layers (mINL) were assessed, excluding asymmetric eyes. PIRA was investigated during ≥ 4 years before the OCT. The associations of retinal layers with PIRA rates were estimated in linear regression adjusted for disease duration, age at onset, sex, body mass index, treatment and annualized relapse rate. In a sensitivity analysis, we investigated the associations between retinal layers and PIRMA rates (PIRA without activity on magnetic resonance imaging).

**Results:**

One hundred seventy one pwMS were included (median age: 51 years(y), Expanded Disability Status Scale: 2.5, pRNFL:94 µm, mGCIPL:67.2 µm, mINL:35.4 µm, observation time:8.1y). Sixty-seven patients (39%) showed PIRA. Mean pRNFL and mGCIPL thickness decreased respectively by − 2.28 µm (95% CI [− 4.32;− 0.24], *p* = 0.029) and − 1.70 µm (95% CI [− 2.97;− 0.42], *p* = 0.010) for each PIRA event per decade, while mINL (beta = − 0.33, CI: [− 0.75;0.1] *p* = 0.133) did not show significant associations with PIRA. In the sensitivity analysis, all three OCT measures were associated with PIRMA (pRNFL: beta = − 3.70, 95% CI [− 6.23; − 1.17], p = 0.005; mGCIPL: beta = − 2.49, 95% CI [− 4.12; − 0.87], *p* = 0.003), mINL: beta = − 0.58, 95% CI [− 1.11; − 0.05], *p* = 0.031).

**Conclusion:**

Our findings underline the role of retinal thinning measured by OCT as sensitive marker of progression in pwMS.

**Supplementary Information:**

The online version contains supplementary material available at 10.1007/s00415-025-13185-y.

## Introduction

Multiple sclerosis (MS) is a chronic disease of the central nervous system (CNS), characterized by inflammatory and neurodegenerative processes [1]. Disability accumulation in MS can result from residual symptoms after relapses and ongoing progression. Recently, the term “PIRA” was introduced to describe progression independent of relapse activity and was shown to be the *main* driver of disability in people with MS (pwMS) [2–4].

PIRA may occur early, is notoriously difficult to capture and quantify, thus could remain undetected or be misclassified by clinicians [2]. Patients with PIRA events within five years after the first demyelinating episode show increased risk for long-term progression [5]. Thus, early identification of PIRA is crucial for the stratification of patients and possibly application of different treatment algorithms. In this regard, magnetic resonance imaging (MRI) markers like cortical atrophy [6], spinal cord atrophy and paramagnetic rim lesions (PRL) [2, 7] as well as serum-biomarkers like glial fibrillary acidic protein (GFAP) [8] were shown to be associated with higher PIRA risk. However, not all these markers have been validated for individual use and are practically applicable.

Optical coherence tomography (OCT) is a non-invasive, highly reproducible method [9] that can quantify neuronal and axonal loss in the retina of pwMS [10]. Retinal atrophy correlates with diffuse gray matter thinning in MS [11] and increased risk of disability worsening [12, 13]. Thus, OCT could offer additional patient-friendly markers of PIRA in MS. Up to date, a single study investigated the association between OCT and PIRA and found an increased rate of retinal atrophy (thinning of the peripapillary retinal nerve fiber layer, pRNFL and the macular ganglion cell-inner plexiform layers, mGCIPL) in patients with PIRA events, over a 4-year period [14].

Our aim was to investigate the relationship between OCT measures (pRNFL, mGCIPL as well as the macular inner nuclear layer, mINL) and the risk of PIRA in a well characterized MS cohort, with longer observation time.

## Methods

### Study participants

We included patients from the MS Center at the University Hospital of Basel (UHB), who participate in the Swiss MS Cohort Study (SMSC), a currently ongoing prospective observational study. Our inclusion criteria were: (1) age ≥ 18 years old (2) MS diagnosis according to the 2017 revised McDonald criteria [15], and (3) at least one available OCT examination. The exclusion criteria were: (1) presence of any retinal pathology that may interfere with the validity of the OCT, as previously described [16], (2) history of both eyes being affected clinically by optic neuritis (ON) (3) less than 4 years of observation time, iv) OCT performed > 6 months apart from the clinical visit.

### Assessment of PIRA rates

Patients were followed longitudinally in the SMSC and assessed every 6–12 months by certified neurologists in the MS Center at the UHB. Physical disability was assessed using the Neurostatus-EDSS instructions and definitions [17]. PIRA events were defined as a relevant increase in the EDSS score compared to the previous (reference) EDSS score, confirmed at a subsequent visit at least 6 months apart and with no clinical relapses between the reference and event visit. The criteria for a relevant EDSS increase were as follows: an increase of ≥ 1.5 points if the baseline EDSS is 0, an increase of ≥ 1 point if the baseline EDSS is between 1.0 and 5, or an increase of ≥ 0.5 points if the baseline EDSS is greater than 5 [4].

We calculated individual PIRA rates, defined as the number of events divided by the total years of observation time per patient.

In addition, we defined PIRMA (Progression Independent of Relapse and MRI Activity) as PIRA events without any new or enlarging lesions on fluid-attenuated inversion recovery (FLAIR) sequences, between reference- and event visits.

### OCT

OCT was performed on a Heidelberg Engineering Spectralis device (Heidelberg, Germany), in a dark room, without pharmacologic pupil dilation. The following OCT markers were assessed on both eyes: (1) mean thickness of the pRNFL, (2) mean thickness of the combined mGCL and mIPL (mGCIPL) and (3) mean thickness of the mINL. Strict quality control of the OCT scans was performed applying the OSCAR-IB criteria [16], which resulted in the exclusion of 37 peripapillary ring scans and 37 macular scans (for details, see supplementary table). Details about the OCT-acquisition settings and scanning protocol according to the APOSTEL 2.0 recommendations [18] are also summarized in Supplementary Table 1.

The pRNFL was measured using 3.4-mm ring scans around the optic nerve head, while the mGCIPL and mINL were measured using a macular volume scan around the fovea (for scan details see supplementary table). After an initial automated segmentation of all intraretinal layers using the software provided by Heidelberg Engineering (Heyex Version 2.5.3), segmentation results of the different layers were checked and corrected where needed by experienced raters. Mean thicknesses of mGCIPL and mINL were calculated using the 6-mm-diameter cylinder of the 1,3,6-mm ring adjacent to the fovea [19] and transforming the volume to mean thickness according to the formula below, where for the 6-mm-diameter cylinder r = 3 mm and thus *π* · *r*^2^ = 28.27433:

$$LayerThickness = \frac{ 1000 \cdot Layer Volume }{{\pi \cdot r^{2} }}$$

$$Layer Thickness = \frac{ 1000 }{{28.27433}} \cdot Layer Volume$$

**Fig. 1:**
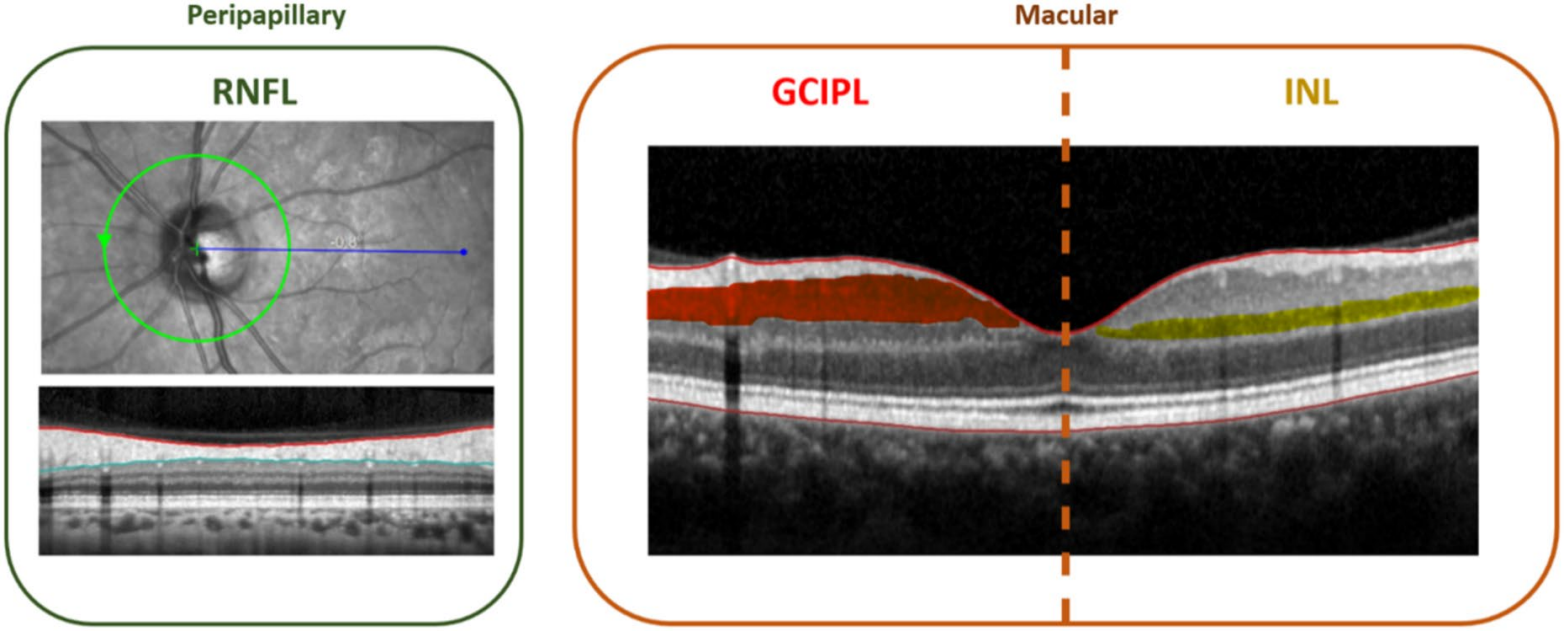
Peripapillary and macular OCT markers used in this study. *GCIPL* ganglion cell-inner plexiform layer, *INL* inner nuclear layer, *OCT* optical coherence tomography, *RNFL* retinal nerve fiber layer

The OCT markers used in the study are also depicted in Fig. 1.

Patients with history of bilateral ON were not included in this study, since the profound bilateral atrophy would mask any meaningful analysis of the subtle atrophy excepted with progressive retinal inner atrophy. In line with this, we also excluded eyes with possible local damage (optic nerve demyelination), by calculating the inter-eye asymmetry, using previously described thresholds: for pRNFL ≥ 5 µm and for mGCIPL ≥ 4 µm [20–22] and excluding the eye with the lower thickness. For patients with asymmetry in mGCIPL, also mINL of the worse eye was excluded from the analysis. For all other patients, without interocular asymmetry, we used the average of right and left layer thicknesses.

### Statistical analysis

All statistical analysis were performed using R [23] version 4.3.3 with packages: ellipsis, readxl, car and ggplot2. Data distributions were analyzed visually and statistically.

To investigate the association between OCT markers and annualized PIRA rates, we used linear regression with each OCT marker as dependent variable and PIRA rate as independent variable (unadjusted models). Then, we performed linear regression models, adjusted for the following characteristics that were previously reported to affect retinal layers and/or PIRA risk: disease duration, age at disease onset, sex, body mass index (BMI), dominant disease modifying treatment (DMT) during the observation time and annualized relapse rate during the observation time. We used the annualized PIRA rates in the years before the OCT examination, to achieve maximum observation time.

Moreover, we performed a sensitivity analysis, using PIRMA (instead of PIRA) rates, with the same variables as described above. Patients were excluded from this analysis if MRI activity could not be assessed for any of their previous PIRA events due to insufficient imaging data (*n* = 22).

We report the estimated coefficients, 95% confidence intervals (CI), and p values for each model; *p* values < 0.05 were considered statistically significant.

## Results

### Patient characteristics

Overall, 171 patients were included. The patients’ characteristics at time of the OCT examination are summarized in Table 1. Most patients were women; while the median disease duration was 16 years, the EDSS was relatively low (median 2.5). Most patients showed a relapsing course and were treated with DMT (Table 1).

**Table 1:** Patient characteristics at time of OCT

Parameter	People with MS (*n* = 171)
Age, median years [IQR]	51.0 [42.4, 59.8]
Sex, female *n* (%)	110 (64.3) %
Disease duration, median years [IQR]	16 [10.5, 23.1]
EDSS, median [IQR]	2.5 [1.5, 4.0]
Relapsing course,* n* (%)	148 (86.5%)
DMT, *n* (%)	145 (84.8%)
Interferon-beta agents	− 8 (4.7%)
Glatirameracetate	− 3 (1.8%)
Fumarates (dimethyl fumarate, diroximel fumarate)	− 14 (8.2%)
Teriflunomide	− 7 (4.1%)
S1P-receptor modulators (fingolimod, ozanimod)	− 60 (35.1%)
Anti-CD20 B-cell-depleting (ocrelizumab, ofatumumab, rituximab)	− 43 (25.2%)
Natalizumab	− 10 (5.8%)
Untreated *n* (%)	26 (15.2%)
pRNFL thickness median [IQR], µm	94.0 [85.8, 102.0]
mGCIPL thickness median [IQR], µm	67.2 [60.1, 70.7]
mINL thickness median [IQR], µm	35.4 [31.8, 35.4]

*DMT* disease modifying Treatment, *EDSS* expanded disability status scale, *mGCIPL* macular ganglion cell-inner plexifom layer, *mINL* macular inner nuclear layer, *IQR* interquartile range, *MS* multiple sclerosis, *OCT* optical coherence tomography, *pRNFL* peripapillary retinal nerve fiber layer

During the follow-up period of at least 4 years before OCT, 67 patients (39%), experienced at least one PIRA event (Table 2).

**Table 2:** PIRA events during the time before OCT

Observation time, years; median [IQR]	8.1 [5.8, 9.4]
Patients with PIRA during observation time (%)	
1 event	55 (32%)
≥ 2 events	12 (7%)

*IQR* interquartile range, *OCT* optical coherence tomography, *PIRA* progression independent of relapse activity

### Associations between OCT markers and annualized PIRA rates

In the unadjusted models, we found associations of pRNFL (beta = − 2.24, 95% CI [− 4.28; − 0.20], *p* = 0.031) as well as mGCIPL (beta = − 1.89, 95% CI [− 3.16; − 0.62], *p* = 0.004) with PIRA rates. In contrast, mINL thickness was not associated with PIRA (beta = − 0.35, 95% CI [− 0.76; 0.06], *p* = 0.092).

**Fig. 2:**
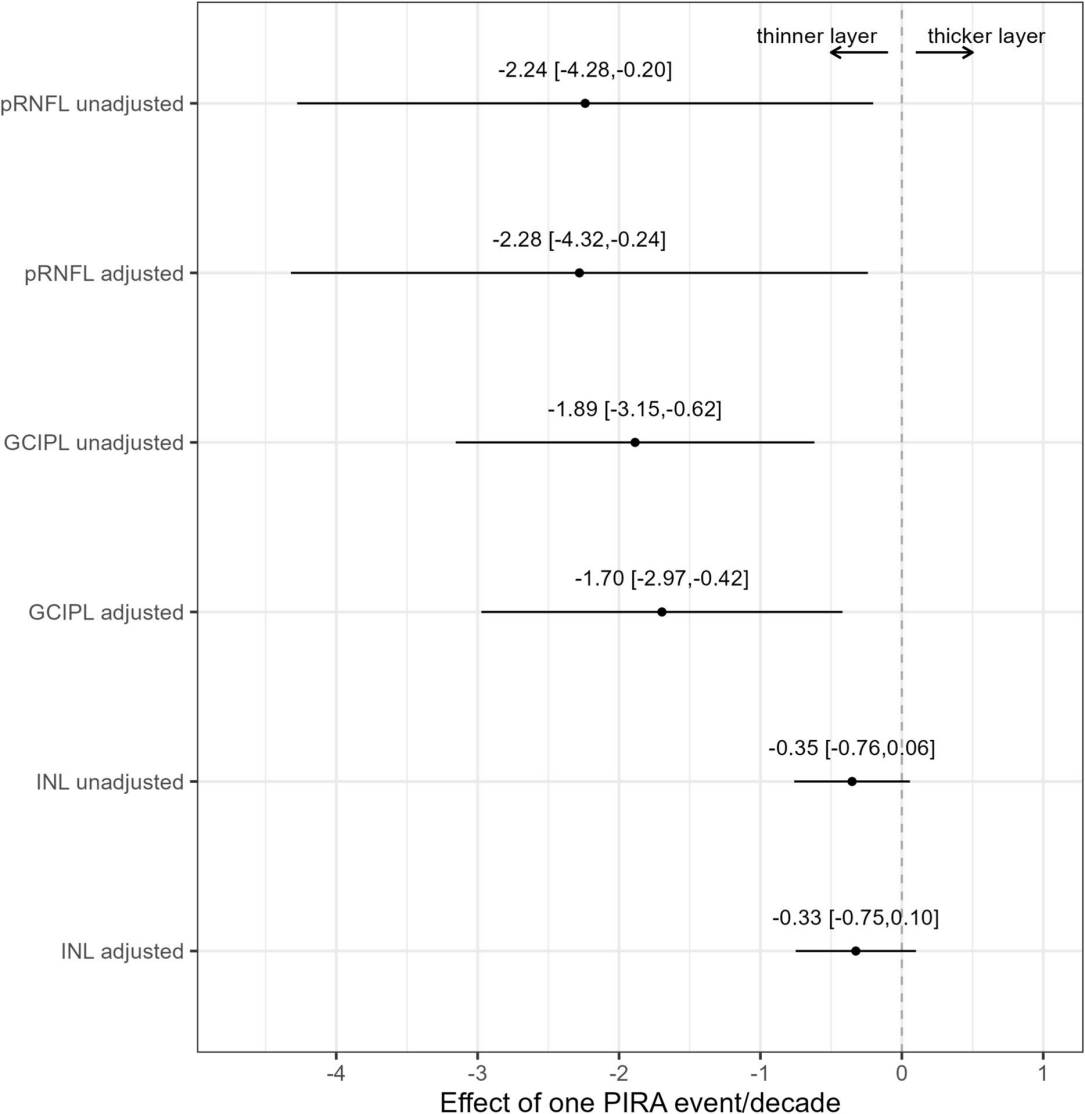
Associations of PIRA rates with the different OCT markers in unadjusted and adjusted models. Forest plot summarizing the results of the linear regression models (unadjusted and fully adjusted for multiple covariates, as described in methods and shown in Table 3). For each OCT measure, the estimated coefficient of PIRA rate (each event per decade) as a point, together with the 95% CI, as a line, are shown. A dashed line at 0 is added. *mGCIPL* macular ganglion cell-inner plexiform layer, *mINL* macular inner nuclear layer, *OCT* optical coherence tomography, *PIRA* progression independent of relapse activity, *pRNFL* peripapillary retinal nerve fiber layer

**Fig. 3:**
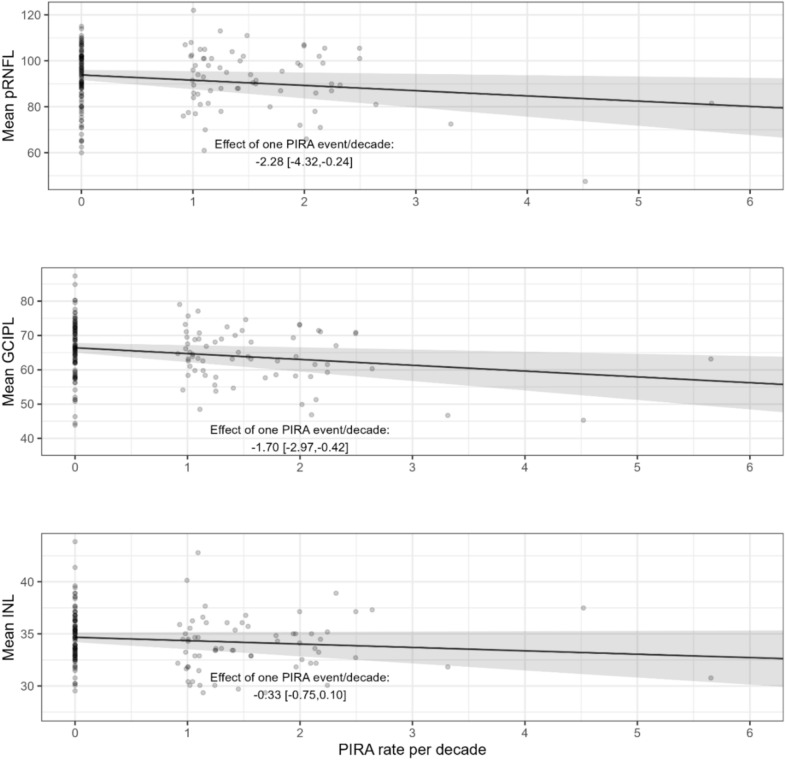
Associations between PIRA rate and OCT markers. Note that the effect sizes of the associations are marginalized over multiple covariates: disease duration, age at onset, sex, body mass index, disease modifying treatment (dominant treatment during observation time) and annualized relapse rate during observation time. Each dot represents the raw data (i.e., combination of OCT parameter and PIRA rate) for a single individual. *mGCIPL* ganglion cell inner plexiform layer (in the macula). *mINL* inner nuclear layer (in the macula), *PIRA* progression independent of relapse activity, *pRNFL* peripapillary retinal nerve fiber layer

In the analysis that was adjusted for multiple patient characteristics (Table 3), the associations of pRNFL and mGCIPL with PIRA rates remained significant. Mean pRNFL thickness decreased by 2.28 µm (95% CI [− 4.32; − 0.24], *p* = 0.029; Table 3). and mean mGCIPL thickness decreased by 1.70 µm for each PIRA event per decade (95% CI [− 2.97; − 0.42], *p* = 0.010; Table 3). Figure 2 summarizes the results of unadjusted and adjusted models for all OCT markers, while Fig. 3 depicts the associations of retinal layers with PIRA rates in the models that were adjusted for multiple characteristics.

**Table 3:** Associations of PIRA rates with the OCT markers, in linear regression models, adjusted for multiple patient characteristics

	pRNFL			mGCIPL			mINL		
	*β*	CI (95%)	*p*	*β*	CI (95%)	*p*	*β*	CI (95%)	*p*
Past PIRA rate per decade	**− 2.28**	**[− 4.32;−0.24]**	**0.029**	**− 1.70**	**[− 2.97; − 0.42]**	**0.010**	− 0.33	[− 0.75;0.1]	0.133
Disease duration	**− 0.47**	**[− 0.67;−0.26]**	** < 0.001**	**− 0.31**	**[− 0.45;−0.17]**	** < 0.001**	− 0.01	[− 0.06;0.03]	0.552
Age at onset	0.11	[−0.10;0.31]	0.304	− 0.04	[− 0.17;0.09]	0.541	− 0.04	[− 0.08;0.01]	0.105
Sex: Female vs. male	0.66	[− 3.27;4.59]	0.740	− 1.57	[− 4.03;0.89]	0.210	− **0.89**	**[**− **1.71;**− **0.07]**	**0.034**
BMI	0.36	[− 0.02;0.75]	0.065	0.17	[− 0.07;0.41]	0.162	0.05	[− 0.03;0.13]	0.255
DMT									
Platform vs. untreated	2.70	[− 5.55;10.95]	0.518	2.20	[− 3.08;7.48]	0.413	− 1.27	[− 3.02;0.49]	0.157
Orals vs. untreated	− 1.50	[− 7.59;4.58]	0.627	0.19	[− 3.66;4.04]	0.923	0.08	[− 1.21;1.36]	0.906
Monoclonals vs. untreated	0.19	[− 6.75;7.13]	0.958	− 0.26	[− 4.72;4.20]	0.909	− 0.23	[− 1.71;1.26]	0.765
Past annualized relapse rate	288.83	[− 969.4;1547.1]	0.651	− 32.18	[− 838.7;774.3]	0.937	26.90	[− 242.9;296.7]	0.844
	R^2^ adj = 0.153			R^2^ adj = 0.156			R^2^ adj = 0.05		

Note that three models were performed, one for each OCT marker. Significant associations are marked in bold. Regarding DMT, platform summarizes interferon beta agents and glatirameracetate, orals summarize S1P receptor modulators, fumarates and teriflunomide, monoclonals summarize B-cell depleting agents and natalizumab (see Table 1)

*β* estimate, *BMI* body mass index, *CI* confidence interval, *DMT* disease modifying treatment, *mGCIPL* macular ganglion cell-inner plexiform layer, *mINL* macular inner nuclear layer, *OCT* optical coherence tomography, *PIRA* progression independent of relapse activity, *pRNFL* peripapillary retinal nerve fiber layer

### Sensitivity analysis with PIRMA rates

Overall, 11 patients with PIRA showed MRI activity between reference and event visits and were thus classified as not having PIRMA. Both pRNFL and GCIPL were associated with PIRMA rates per decade in the adjusted models (pRNFL: beta = − 3.70, 95% CI [− 6.23; − 1.17], *p* = 0.005; mGCIPL: beta = − 2.49, 95% CI [− 4.12; − 0.87], *p* = 0.003) (Supplementary Table 1). Interestingly, also mINL thickness was associated with PIRMA (beta = − 0.58, 95% CI [− 1.11; − 0.05], *p* = 0.031, Supplementary Table 2).

## Discussion

We investigated the relationship between retinal layers and PIRA in a well characterized MS cohort with long observation time (median of approximately 8 years). Our main finding is, that CNS-neuronal and axonal loss in the retina, as reflected by thinner mGCIPL and pRNFL, is associated with higher PIRA rates. In contrast, mINL did not show associations with PIRA. The relationship between pRNFL and mGCIPL with PIRA remained, after adjustment for multiple patient characteristics, including disease duration, DMT and annualized relapse rates.

Associations between OCT markers and PIRA were previously described in a single study [13], which reported that patients experiencing PIRA events showed higher rates of pRNFL- and mGCIPL thinning over 4 years. Our results are in line with these prior findings and confirm an association between OCT and PIRA over a longer observation time.

Interestingly, a recent multicenter study [29] found a significant association between thinning of the pRNFL and higher likelihood of relapses among patients with RRMS, while mGCIPL was not associated with clinical disease activity [29]. We found that the relationship between both OCT measures and PIRA remained after adjustment for characteristics that are related to inflammatory activity, like annualized relapse rate and DMT. In addition, mGCIPL thinning was related to subclinical (MRI) disease activity in the previous multicenter study [29], underlying the complex relationship between retinal thinning, inflammatory- and neurodegenerative processes in MS. In our sensitivity analysis, we still found significant associations between both OCT measures (pRNFL, mGCIPL) and PIRMA events. This suggests that the relationship between retinal layers and PIRA was not driven by subclinical inflammatory activity in our study. Taking into account the high reproducibility of OCT [9] and the patient-friendly, quick implementation, our findings support the role of mGCIPL and pRNFL as useful clinical markers of progression independent of disease activity in pwMS.

It has to be noted, that although both mGCIPL and pRNFL were associated with PIRA and PIRMA in our study, mGCIPL may be less prone to axonal swelling and inflammatory processes in the visual pathway and may thus be a more specific marker for neurodegeneration in MS [25–28]. Thus, it is probably meaningful to include macular —not only peripapillary— scans in the OCT protocol, when aiming at capturing neurodegenerative processes and PIRA. The macular scans have also the advantage of less anatomic variability than the papillary/optic-disk area [30].

Compared to the layers containing the axons (pRNFL) and CNS-neurons (GCL/GCIPL) of the retina, the role of the mINL as a marker of clinical disease activity or progression in MS is less understood. In a prior longitudinal multicenter study, mINL thickening was shown to be indicative of adjacent optic nerve inflammation, as well as to correlate with the occurrence of relapses not involving the optic nerve, but not with increase of disability over time [31]. Others showed that high mINL volume may be associated with MRI- and clinical inflammatory activity [24, 31–33]. A recent multicenter study suggested that mINL thickening may occur in the early and more inflammatory stages in RRMS, followed by mINL atrophy in later stages or progressive forms of MS [29]. We did not find an association of mINL with PIRA in our study, although our patients had overall a long disease duration. However, in the sensitivity analysis taking MRI activity into account, thinner mINL was indeed associated with PIRMA rates. This suggests that subclinical inflammatory activity may affect the INL thickness in certain patients, thereby “masking” an association between mINL thinning and PIRA in the entire group. Due to this “double” role of mINL, and based on all our results, pRNFL and mGCIPL seem more suitable OCT markers than mINL to reflect progression in pwMS.

Of note, we found almost no associations between patient characteristics like sex, BMI and DMT and retinal layers in our multivariate models, although this could be due to the stronger effects of PIRA and disease duration on retinal thinning. The negative association between disease duration and retinal layers (pRNFL and mGCIPL) is in line with previous reports [29, 34].

It is also interesting to comment on the proportion of patients with PIRA in our study: 39% experienced at least one PIRA event during 8 years of observation time, while, as expected [35], the proportion was higher in patients with progressive disease courses (78% vs. 33% in patients having officially a relapsing course). Our PIRA rates are overall slightly higher than previously reported (e.g., 25% in a study with median follow-up of approximately 7 years [5] and 28% in another study with follow-up of approximately 12 years [2]). This could be attributed to differences among the study populations or slightly modified PIRA definitions used in the different studies. Noteworthy, we applied a relatively strict definition of PIRA, not allowing any relapses between reference- and event visits. Overall, the high incidence of PIRA in our- and previous studies highlights its relevance as main driver of disability in pwMS [2, 4].

Strengths of our study are the long clinical observation period of approximately 8 years (compared to the previous shorter study [14]), as well as the well characterized large MS cohort, including accurate assessment of disability every 6–12 months, based on the Neurostatus-EDSS definitions and information on MRI activity for most patients. Regarding limitations, this study was monocentric and included predominantly white pwMS, which may limit the generalizability of our results. We did not include healthy control data in this analysis, however previous studies already demonstrated that the rate of atrophy in retinal layers is significantly lower in healthy individuals compared to pwMS [36, 37]. We evaluated PIRA events in time *before* OCT, since this significantly increased the length of observation time and the number of PIRA events. Since the assessment of disability and definition of PIRA events were performed prospectively, we do not believe that this is a major drawback. Prospective studies, using OCT to predict future risk of PIRA, including multi-ethnic population and combination with other biomarkers are needed to advance our understanding of PIRA in MS and establish the utility of different measures for patient stratification. Furthermore, prospective diagnosis of ON, according to recently published criteria, which also included the rare form of primary progressive ON [20], as a “pure” form of PIRA of the optic nerve, would contribute to our understanding of the complex relationship between the visual system and progression in pwMS.

To conclude, our findings underline the role of OCT-based neuronal- and axonal loss in the CNS (mGCIPL, pRNFL) as sensitive, patient-friendly markers of PIRA and PIRMA. Prospective larger studies are needed to define the relative value of retinal biomarkers in patient stratification and prognostic algorithms in pwMS.

## Supplementary Information

Below is the link to the electronic supplementary material.

Supplementary file1 (DOCX 33 KB)

Supplementary file2 (DOCX 21 KB)

## Data Availability

Fully anonymized (encoded) data of this study could become available from the corresponding author, upon reasonable request from qualified investigators.
