# Supplementary material for: Retinal neuronal loss and progression independent of relapse activity in multiple sclerosis
Source: J Neurol. 2025 Jun 10;272(7):454. doi: 10.1007/s00415-025-13185-y (PMC12152092; doi:10.1007/s00415-025-13185-y)
Supplement: Supplementary file 1 — Supplementary file1 (DOCX 33 KB) [file 415_2025_13185_MOESM1_ESM.docx]

**Supplementary table 1: Summary of the 9 APOSTEL 2.0 items regarding the Optical Coherence Tomography (OCT-) data of this study** (1)**.**

| **Item** | | **Description** |
| --- | --- | --- |
| 1 | Study protocol | Monocentric study in the Neurology Department of the University Hospital of Basel. The OCT examination had a duration of approximately 10-15 minutes and was usually performed on the same day as the assessment of the EDSS (if not, within six months).  Study inclusion criteria: i) age ≥ 18 years old ii) MS diagnosis according to the 2017 revised McDonald criteria (2), and iii) at least one available OCT examination within the Swiss MS Cohort study  Study exclusion criteria: i) presence of any retinal pathology that may interfere with the validity of the OCT, as previously described in the OSCAR-IB criteria (3), ii) history of both eyes being affected clinically by ON iii) clinical observation time < 4 years, iv) OCT performed > 6 months apart from the clinical visit. |
| 2 | Acquisition device | Heidelberg Engineering Spectralis (Heidelberg, Germany)  Software version: Eye Explorer Heidelberg Engineering (Heyex), Version 2.5.3  Device type: spectral domain (SD) OCT |
| 3 | Acquisition settings | Pupils were not pharmacologically dilated, but OCT was performed in a dark room. A single device was used, by three previously trained OCT-operators with excellent inter-rater agreement. |
| 4 | Scanning protocol | The scanning protocol included a circular scan around the optic disc and a volume scan from the macula centered on the fovea, both with the eye tracking function enabled.  i) The details of the circular (ring) scan for the assessment of the pRNFL thickness are: 12°, 1536 A-scans, ART between 12 and 100, high resolution (HR) setting, manual placement of the ring  ii) The details of the macular volume scan, for the assessment of the mGCIPL and mINL thicknesses were: 25°x30°, 61 vertically oriented B-scans, 768 A-scans per B-scan, ART between 13 and 17, 768x496 pixels for each B-scan, high speed (HS) setting |
| 5 | Fundoscopy | No fundus photography included in this study. |
| 6 | Post-acquisition data selection | Strict quality control of the OCT scans, using the validated OSCAR-IB criteria (3), which resulted in the exclusion of the following number of scans: peripapillary circular scans from 37 eyes (n=10 due to technical reasons and n= 27 due to incidental findings) and macular volume scans from 37 eyes (n= 17 due to technical reasons and n=20 due to incidental findings).  Eye-selection strategy: We calculated the inter-eye asymmetry, using previously described thresholds: for pRNFL ≥ 5 μm (4) and for mGCIPL ≥ 4 μm (5). In case of interocular asymmetry, we excluded the eye with the thinner layer from the analysis (n= 42 for peripapillary scans and n= 9 for macular scans). In case of symmetric values, we used the average of both eyes in the analysis. |
| 7 | Post-acquisition analysis | Automated segmentation of the macular GCL, IPL and INL, as well as peripapillary RNFL using the software provided by the manufacturer (Heyex Version 2.5.3, Heidelberg Engineering)  All scans were checked by two experienced OCT-raters and manually corrected where needed, especially in the outer ring area.  Mean thicknesses of mGCIPL and mINL were calculated using the 6-mm-diameter cylinder of the 1,3,6 mm ring adjacent to the fovea and transforming the volume to mean thickness according to the formula: thickness = volume / 0.02827433, where the constant derives from the cylindrical geometry of the ETDRS grid with a 1-, 3- and 6-mm diameter (radius = 3mm). For the combined mGCIPL volume, the volumes of the ganglion cell layer and the inner plexiform layer were added for each eye. |
| 8 | Nomenclature and abbreviations | Nomenclature compatible with Figure 1 in APOSTEL 2.0 guidelines:  - pRNFL: peripapillary retinal nerve fiber layer (µm) - mGCIPL: macular ganglion cell- inner plexiform layer (µm)  - mINL: macular inner nuclear layer (µm) |
| 9 | Statistical approach | The analysis was performed per patient, due to the aim (association with PIRA, which is a per-patient event). Thus, we considered the average thicknesses of both eyes for the statistical analysis. However, in case of interocular asymmetry, we excluded the eye with the thinner layer, as described above. Linear regression analysis was performed using R, as described in Methods. |

Abbreviations: ART: automatic real-time tracking; EDSS: expanded disability status scale; mGCIPL: ganglion cell inner plexiform layer; mINL: inner nuclear layer; OCT: optical coherence tomography; ON: optic neuritis; PIRA: progression independent of relapse activity; pRNFL: peripapillary retinal nerve fiber layer.

References:

1. Aytulun A, Cruz-Herranz A, Aktas O, Balcer LJ, Balk L, Barboni P, et al. APOSTEL 2.0 Recommendations for Reporting Quantitative Optical Coherence Tomography Studies. Neurology. 2021 Jul 13;97(2):68–79.

2. Thompson AJ, Banwell BL, Barkhof F, Carroll WM, Coetzee T, Comi G, et al. Diagnosis of multiple sclerosis: 2017 revisions of the McDonald criteria. Lancet Neurol. 2018 Feb;17(2):162–73.

3. Tewarie P, Balk L, Costello F, Green A, Martin R, Schippling S, et al. The OSCAR-IB Consensus Criteria for Retinal OCT Quality Assessment. PLOS ONE. 2012 Apr 19;7(4):e34823.

4. Nolan RC, Galetta SL, Frohman TC, Frohman EM, Calabresi PA, Castrillo-Viguera C, et al. Optimal Intereye Difference Thresholds in Retinal Nerve Fiber Layer Thickness for Predicting a Unilateral Optic Nerve Lesion in Multiple Sclerosis. J Neuro-Ophthalmol Off J North Am Neuro-Ophthalmol Soc. 2018 Dec;38(4):451–8.

5. Petzold A, Chua SYL, Khawaja AP, Keane PA, Khaw PT, Reisman C, et al. Retinal asymmetry in multiple sclerosis. Brain. 2021 Jan 1;144(1):224–35.
