# Supplementary material for: Retinal neuronal loss and progression independent of relapse activity in multiple sclerosis
Source: J Neurol. 2025 Jun 10;272(7):454. doi: 10.1007/s00415-025-13185-y (PMC12152092; doi:10.1007/s00415-025-13185-y)
Supplement: Supplementary file 2 — Supplementary file2 (DOCX 21 KB) [file 415_2025_13185_MOESM2_ESM.docx]

|  | **pRNFL** | | | **mGCIPL** | | | **mINL** | | |
| --- | --- | --- | --- | --- | --- | --- | --- | --- | --- |
|  | **β** | **CI (95%)** | **P** | **β** | **CI (95%)** | **P** | **β** | **CI (95%)** | **P** |
| **Past PIRMA rate per decade** | **-3.70** | **[-6.23;-1.17]** | **0.005** | **-2.49** | **[-4.12;-0.87]** | **0.003** | **-0.58** | **[-1.11;-0.05]** | **0.031** |
| Disease duration | **-0.38** | **[-0.61;-0.16]** | **0.001** | **-0.29** | **[-0.43;-0.14]** | **<0.001** | -0.01 | [-0.06;0.04] | 0.594 |
| Age at onset | 0.12 | [-0.10;0.33] | 0.289 | -0.01 | [-0.15;0.13] | 0.884 | -0.03 | [-0.07;0.02] | 0.218 |
| Sex:  Female vs. male | 2.01 | [-2.15;6.18] | 0.340 | -1.28 | [-3.95;1.39] | 0.344 | **-1.20** | **[-2.07;-0.34]** | **0.007** |
| BMI | 0.37 | [-0.03;0.76] | 0.071 | 0.18 | [-0.08;0.44] | 0.167 | 0.04 | [-0.05;0.12] | 0.399 |
| DMT: |  | | |  | | |  | | |
| - Platform vs. untreated | -0.63 | [-9.90;8.64] | 0.893 | 0.93 | [-5.16;7.02] | 0.763 | -1.85 | [-3.82;0.12] | 0.066 |
| - Orals vs. untreated | -2.80 | [-9.97;4.38] | 0.442 | -0.29 | [-4.93;4.35] | 0.902 | -0.58 | [-2.09;0.93] | 0.446 |
| - Monoclonals vs. untreated | -0.08 | [ -8.00;7.84] | 0.984 | -0.52 | [-5.67;4.62] | 0.841 | -0.66 | [-2.33;1.00] | 0.433 |
| Past annualised relapse rate | 410.5 | [-903.2; 1724.2] | 0.538 | 139.6 | [-717.5; 996.7] | 0.748 | 79.3 | [-199.4;358] | 0.575 |
|  | R ^2^ adj=0.128 | | | R ^2^ adj = 0.142 | | | R ^2^ adj = 0.069 | | |

**Supplementary Table 2: Associations of PIRMA rates per decade with the OCT markers, in linear regression models, adjusted for multiple patient characteristics**

Legend Table S1: Note that three models were performed, one for each OCT marker. PIRMA was defined as PIRA event without any signs of MRI activity between event- and reference visits. Significant associations are marked in bold. Regarding DMT, platform summarizes interferon beta agents and glatirameracetate, orals summarize S1P receptor modulators, fumarates and teriflunomide, monoclonals summarize B-cell depleting agents and natalizumab.

Abbreviations: β: estimate; BMI: body mass index; CI: confidence interval; DMT: disease modifying treatment; mGCIPL: macular ganglion cell inner plexiform layer; mINL: macular inner nuclear layer; MRI: magnetic resonance imaging; OCT: optical coherence tomography; PIRMA: progression independent of relapse and MRI activity; pRNFL: peripapillary retinal nerve fiber layer.
